# ARIA‐EAACI care pathways for allergen immunotherapy in respiratory allergy

**DOI:** 10.1002/clt2.12014

**Published:** 2021-06-09

**Authors:** Jean Bousquet, Oliver Pfaar, Ioana Agache, Anna Bedbrook, Cezmi A Akdis, G. Walter Canonica, Tomas Chivato, Mona Al‐Ahmad, Amir H Abdul Latiff, Ignacio J Ansotegui, Claus Bachert, Abdullah Baharuddin, Karl‐Christian Bergmann, Carsten Bindslev‐Jensen, Leif Bjermer, Matteo Bonini, Sinthia Bosnic‐Anticevich, Isabelle Bosse, Helen A. Brough, Luisa Brussino, Moises A Calderon, Luis Caraballo, Victoria Cardona, Pedro Carreiro‐Martins, Tomas Casale, Lorenzo Cecchi, Alfonso M Cepeda Sarabia, Ekaterine Chkhartishvili, Derek K Chu, Ieva Cirule, Alvaro A Cruz, Wienczyslawa Czarlewski, Stefano del Giacco, Pascal Demoly, Philippe Devillier, Dejan Dokic, Stephen L Durham, Motohiro Ebisawa, Yehia El‐Gamal✝, Regina Emuzyte, Amiran Gamkrelidze, Jean Luc Fauquert, Alessandro Fiocchi, Wytske J Fokkens, Joao A Fonseca, Jean‐François Fontaine, Radoslaw Gawlik, Asli Gelincik, Bilun Gemicioglu, Jose E Gereda, Roy Gerth van Wijk, R Maximiliano Gomez, Maia Gotua, Ineta Grisle, Maria‐Antonieta Guzmán, Tari Haahtela, Susanne Halken, Enrico Heffler, Karin Hoffmann‐Sommergruber, Elham Hossny, Martin Hrubiško, Carla Irani, Juan Carlos Ivancevich, Zhanat Ispayeva, Kaja Julge, Igor Kaidashev, Omer Kalayci, Musa Khaitov, Ludger Klimek, Edward Knol, Marek L Kowalski, Helga Kraxner, Inger Kull, Piotr Kuna, Violeta Kvedariene, Vicky Kritikos, Antti Lauerma, Susanne Lau, Daniel Laune, Michael Levin, Desiree E Larenas‐Linnemann, Karin C Lodrup Carlsen, Carlo Lombardi, Olga M Lourenço, Bassam Mahboub, Hans‐Jørgen Malling, Patrick Manning, Gailen D Marshall, Erik Melén, Eli O Meltzer, Neven Miculinic, Branislava Milenkovic, Mostafa Moin, Stephen Montefort, Mario Morais‐Almeida, Charlotte G Mortz, Ralph Mösges, Joaquim Mullol, Leyla Namazova Baranova, Hugo Neffen, Kristof Nekam, Marek Niedoszytko, Mikaëla Odemyr, Robyn E O'Hehir, Markus Ollert, Liam O'Mahony, Ken Ohta, Yoshitaka Okamoto, Kimi Okubo, Giovanni B Pajno, Oscar Palomares, Susanna Palkonen, Petr Panzner, Nikolaos G Papadopoulos, Hae‐Sim Park, Giovanni Passalacqua, Vincenzo Patella, Ruby Pawankar, Nhân Pham‐Thi, Davor Plavec, Todor A Popov, Marysia Recto, Frederico S Regateiro, Carmen Riggioni, Graham Roberts, Monica Rodriguez‐Gonzales, Nelson Rosario, Menachem Rottem, Philip W Rouadi, Dermot Ryan, Boleslaw Samolinski, Mario Sanchez‐Borges✝, Faradiba S Serpa, Joaquin Sastre, Glenis K. Scadding, Mohamed H Shamji, Peter Schmid‐Grendelmeier, Holger J Schünemann, Aziz Sheikh, Nicola Scichilone, Juan Carlos Sisul, Mikhail Sofiev, Dirceu Solé, Talant Sooronbaev, Manuel Soto‐Martinez, Manuel Soto‐Quiros, Milan Sova, Jürgen Schwarze, Isabel Skypala, Charlotte Suppli‐Ulrik, Luis Taborda‐Barata, Ana Todo‐Bom, Maria J Torres, Marylin Valentin‐Rostan, Peter‐Valentin Tomazic, Antonio Valero, Sanna Toppila‐Salmi, Ioanna Tsiligianni, Eva Untersmayr, Marilyn Urrutia‐Pereira, Arunas Valiulis, Erkka Valovirta, Olivier Vandenplas, Maria Teresa Ventura, Pakit Vichyanond, Martin Wagenmann, Dana Wallace, Jolanta Walusiak‐Skorupa, De Yun Wang, Susan Waserman, Gary WK Wong, Arzu Yorgancioglu, Osman M Yusuf, Mario Zernotti, Luo Zhang, Mihaela Zidarn, Torsten Zuberbier, Marek Jutel

**Affiliations:** ^1^ Department of Dermatology and Allergy Comprehensive Allergy Center Charité Universitätsmedizin Berlin Humboldt‐Universität zu Berlin, and Berlin Institute of Health Berlin Germany; ^2^ Allergology University Hospital Montpellier Montpellier France; ^3^ MACVIA‐France Montpellier France; ^4^ Department of Otorhinolaryngology Head and Neck Surgery Section of Rhinology and Allergy University Hospital Marburg Philipps‐Universität Marburg Germany; ^5^ Allergy and Clinical Immunology Transylvania University Brasov Brasov Romania; ^6^ Swiss Institute of Allergy and Asthma Research (SIAF) University of Zurich Davos Switzerland; ^7^ Department of Biomedical Sciences Humanitas University Pieve Emanuele (MI) and Personalized Medicine, Asthma and Allergy, Humanitas Clinical and Research Center IRCCS, Milano Italy; ^8^ School of Medicine University CEU San Pablo Madrid, Spain; ^9^ Department of Allergy and Microbiology Faculty of Medicine Al‐Rashed Allergy Center Kuwait University Kuwait City Kuwait; ^10^ Allergy & Immunology Centre Pantai Hospital Kuala Lumpur Malaysia; ^11^ Department of Allergy and Immunology Hospital Quironsalud Bizkaia Erandio, Spain; ^12^ ENT Department Upper Airways Research Laboratory Ghent University Hospital Ghent Belgium; ^13^ Department of Otorhinolaryngology—Head and Neck Surgery School of Medical Sciences Universiti Sains Malaysia Kubang Kerian Kelantan Malaysia; ^14^ Department of Dermatology and Allergy Centre Odense University Hospital Odense Denmark; ^15^ Department of Respiratory Medicine and Allergology University Hospital Lund Sweden; ^16^ Department of Cardiovascular and Thoracic Sciences Fondazione Policlinico Universitario A Gemelli IRCCS Università Cattolica del Sacro Cuore Rome Italy; ^17^ Woolcock Institute of Medical Research University of Sydney Australia; ^18^ Allergist La Rochelle France; ^19^ Paediatric Allergy Department of Asthma, Allergy and Respiratory Science Guys' Hospital King's College London London UK; ^20^ Department of Medical Sciences Allergy and Clinical Immunology Unit University of Torino & Mauriziano Hospital Torino Italy; ^21^ Imperial College and National Heart and Lung Institute London UK; ^22^ Institute for Immunological Research University of Cartagena Cartagena Colombia; ^23^ Allergy Section Department of Internal Medicine Hospital Vall d'Hebron & ARADyAL Research Network Barcelona, Spain; ^24^ Serviço de Imunoalergologia Hospital de Dona Estefânia Centro Hospitalar de Lisboa Central Lisbon Portugal; ^25^ Division of Allergy/immunology University of South Florida Tampa Fla USA; ^26^ SOS Allergology and Clinical Immunology USL Toscana Centro Prato Italy; ^27^ Allergy and Immunology Laboratory Metropolitan University Simon Bolivar University Barranquilla Colombia; ^28^ David Tatishvili Medical Center David Tvildiani Medical University‐AIETI Medical School Tbilisi Georgia; ^29^ Departments of Medicine and Health Research Methods McMaster University Hamilton ON Canada; ^30^ Latvain Association of Allergists University Children Hospital Riga Latvia; ^31^ Fundação ProAR Federal University of Bahia and GARD/WHO Planning Group Salvador Brazil; ^32^ Medical Consulting Czarlewski Levallois France; ^33^ Department of Medical Sciences and Public Health and Unit of Allergy and Clinical Immunology University Hospital “Duilio Casula” University of Cagliari Cagliari Italy; ^34^ Department of Pulmonology Division of Allergy Hôpital Arnaud de Villeneuve University Hospital of Montpellier France; ^35^ Unité de Recherche en Pharmacologie Respiratoire Pôle des Maladies des Voies Respiratoires, Hôpital Foch Université Paris Saclay Suresnes France; ^36^ Medical Faculty University Clinic of Pulmology and Allergy Skopje Republic of Macedonia; ^37^ National Heart and Lung Institute Imperial College London UK; ^38^ Clinical Reserch Center for Allergy and Rheumatology NHO Sagamihara National Hospital Sagamihara Japan; ^39^ Pediatric Allergy and Immunology Unit Children's Hospital Ain Shams University Cairo Egypt; ^40^ Faculty of Medicine Clinic of Children's Diseases Vilnius University Vilnius Lithuania; ^41^ National Center for Disease Control and Public Health of Georgia Tbilisi Georgia; ^42^ CHU Clermont‐Ferrand Unité d'Allergologie de l'Enfant Pole pédiatrique Hopital Estaing Clermont‐Ferrand France; ^43^ Division of Allergy The Bambino Gesu Children's Hospital IRCCS Rome Italy; ^44^ Department of Otorhinolaryngology Academic Medical Centers Amsterdam The Netherland; ^45^ Faculdade de Medicina CINTESIS, Center for Health Technology and Services Research Universidade do Porto Porto Portugal; ^46^ Allergist Reims France; ^47^ Department of Internal Medicine, Allergology and Clinical Immunology Silesian University of Medicine Katowice Poland; ^48^ Division of Allergy and Immunology Dışkapı Yıldırım Beyazıt Training and Research Hospital Ankara Turkey; ^49^ Department of Pulmonary Diseases Cerrahpasa Faculty of Medicine Istanbul University‐Cerrahpasa Istanbul Turkey; ^50^ Allergy and Immunology Division Clinica Ricardo Palma Lima Peru; ^51^ Department of Internal Medicine Section of Allergology Erasmus MC Rotterdam the Netherlands; ^52^ Fundacion Ayre Instituto Medico Alas Salta Argentina; ^53^ Center of Allergy and Immunology Georgian Association of Allergology and Clinical Immunology Tbilisi Georgia; ^54^ Latvian Association of Allergists Center of Tuberculosis and Lung Diseases Riga Latvia; ^55^ Immunology and Allergy Division Clinical Hospital University of Chile Santiago Chile; ^56^ Skin and Allergy Hospital Helsinki University Hospital Helsinki Finland; ^57^ Hans Christian Andersen Children's Hospital Odense University Hospital Odense Denmark; ^58^ Department of Pathophysiology and Allergy Research Medical University of Vienna Vienna Austria; ^59^ Pediatric Allergy and Immunology Unit Children's Hospital Ain Shams University Cairo Egypt; ^60^ Department of Clinical Immunology and Allergy Oncology Institute of St Elisabeth Bratislava Slovakia; ^61^ Department of Internal Medicine and Infectious Diseases St Joseph University Hotel Dieu de France Hospital Beirut Lebanon; ^62^ Servicio de Alergia e Immunologia Clinica Santa Isabel Buenos Aires Argentina; ^63^ Department of Allergology and Clinical Immunology of the Kazakh National Medical University Kazakhstan Association of Allergology and Clinical Immunology Kazakhstan; ^64^ Allergy Center of Childrens's Clinic of Tartu University Hospital Tartu Estonia; ^65^ Ukrainina Medical Stomatological Academy Poltava Ukraine; ^66^ Pediatric Allergy and Asthma Unit Hacettepe University School of Medicine Ankara Turkey; ^67^ National Research Center Institute of Immunology Federal Medicobiological Agency Laboratory of Molecular Immunology Russia; ^68^ Department of Otolaryngology Head and Neck Surgery Universitätsmedizin Mainz Mainz Germany; ^69^ Departments of Immunology and Dermatology/Allergology University Medical Center Utrecht The Netherlands; ^70^ Department of Immunology and Allergy Healthy Ageing Research Center Medical University of Lodz Poland; ^71^ Department of Otorhinolaryngology Head and Neck Surgery Semmelweis University Budapest Hungary; ^72^ Department of Clinical Science and Education Södersjukhuset Karolinska Institutet Stockholm Sweden; ^73^ Division of Internal Medicine, Asthma and Allergy Barlicki University Hospital Medical University of Lodz Poland; ^74^ Department of Pathology Faculty of Medicine Institute of Biomedical Sciences Vilnius University Vilnius Lithuania; ^75^ Quality Use of Respiratory Medicines Group Woolcock Institute of Medical Research University of Sydney Sydney NSW Australia; ^76^ Department of Dermatology and Allergology University of Helsinki and Helsinki University Helsinki Finland; ^77^ Department of Pediatric Pneumology and Immunology Charité Universitätsmedizin Berlin Germany; ^78^ KYomed INNOV Montpellier France; ^79^ Division of Paediatric Allergology University of Cape Town Cape Town South Africa; ^80^ Center of Excellence in Asthma and Allergy Médica Sur Clinical Foundation and Hospital México City Mexico; ^81^ Department of Paediatrics Oslo University Hospital Oslo Norway; ^82^ Departmental Unit of Allergology & Respiratory Diseases Fondazione Poliambulanza Brescia Italy; ^83^ Faculty of Health Sciences and CICS – UBI Health Sciences Research Centre University of Beira Interior Covilhã Portugal; ^84^ Department of Pulmonary Medicine Rashid Hospital Dubai UAE; ^85^ Danish Allergy Centre University of Copenhagen Copenhagen Denmark; ^86^ Department of Medicine (RCSI) Bon Secours Hospital Dublin Ireland; ^87^ The University of Mississippi Medical Center Division of Clinical Immunology and Allergy Laboratory of Behavioral Immunology Research Jackson Mississippi USA; ^88^ Sachs' Children and Youth Hospital Södersjukhuset Stockholm Sweden; ^89^ Allergy Allergy and Asthma Medical Group and Research Center San Diego California USA; ^90^ Croatian Pulmonary Society Zagreb Croatia; ^91^ Faculty of Medicine Clinic for Pulmonary Diseases Clinical Center of Serbia University of Belgrade Belgrade Serbia; ^92^ Immunology and Asthma and Allergy Research Institute Tehran University of Medical Sciences Tehran Iran; ^93^ Faculty of Medicine and Surgery Mater Dei Hospital Malta University of Medicine La Valette Malta; ^94^ Allergy Center CUF Descobertas Hospital Lisbon Portugal; ^95^ CRI‐Clinical Research International‐Ltd Hamburg Germany; ^96^ ENT Department Rhinology Unit & Smell Clinic Hospital Clínic Barcelona Spain; ^97^ Scientific Centre of Children's Health Russian National Research Medical University Moscow Russia; ^98^ Center of Allergy, Immunology and Respiratory Diseases Santa Fe Argentina; ^99^ Hospital of the Hospitaller Brothers in Buda Budapest Hungary; ^100^ Department of Allergology Medical University of Gdańsk Gdańsk Poland; ^101^ EFA European Federation of Allergy and Airways Diseases Patients' Associations Brussels Belgium; ^102^ Department of Allergy, Immunology and Respiratory Medicine Central Clinical School Monash University Victoria Australia; ^103^ Department of Infection and Immunity Luxembourg Institute of Health Esch‐sur‐Alzette Luxembourg; ^104^ Departments of Medicine and Microbiology APC Microbiome Ireland University College Cork Cork Ireland; ^105^ National Hospital Organization Tokyo National Hospital Tokyo Japan; ^106^ Department of Otorhinolaryngology Chiba University Hospital Chiba Japan; ^107^ Department of Otolaryngology Nippon Medical School Tokyo Japan; ^108^ Department of Pediatrics Allergy Unit University of Messina Messina Italy; ^109^ Department of Biochemistry and Molecular Biology School of Chemistry Complutense University of Madrid Madrid, Spain; ^110^ Department of Immunology and Allergology Faculty of Medicine and Faculty Hospital in Pilsen Charles University in Prague Pilsen Czech Republic; ^111^ Division of Infection Immunity & Respiratory Medicine Royal Manchester Children's Hospital University of Manchester Manchester UK; ^112^ Department of Allergy and Clinical Immunology Ajou University School of Medicine Suwon South Korea; ^113^ Allergy and Respiratory Diseases Ospedale Policlino San Martino ‐University of Genoa Italy; ^114^ Department of Medicine Division of Allergy and Clinical Immunology Agency of Health ASL Salerno “Santa Maria della Speranza” Hospital Battipaglia Salerno Italy; ^115^ Department of Pediatrics Nippon Medical School Tokyo Japan; ^116^ Ecole Polytechnique Palaiseau IRBA (Institut de Recherche bio‐Médicale des Armées) Bretigny France; ^117^ School of Medicine Children's Hospital Srebrnjak, Zagreb University J.J. Strossmayer Osijek Croatia; ^118^ University Hospital ‘Sv Ivan Rilski’ Sofia Bulgaria; ^119^ Allergy and Clinical Immunology Unit Centro Hospitalar e Universitário de Coimbra Coimbra Coimbra Portugal; ^120^ Pediatric Allergy and Clinical Immunology Department Hospital Sant Joan de Déu Barcelona Spain; ^121^ Salford Royal NHS Foundation Trust NHS England North Salford UK; ^122^ Pediatric Allergy and Clinical Immunology Hospital Angeles Pedregal Mexico City Mexico; ^123^ Hospital de Clinicas University of Parana Brazil; ^124^ Division of Allergy Asthma and Clinical Immunology Emek Medical Center Afula Israel; ^125^ Department of Otolaryngology‐Head and Neck Surgery Eye and Ear University Hospital Beirut Lebanon; ^126^ Usher Institute Medical School University of Edinburgh Edinburgh UK; ^127^ Department of Prevention of Environmental Hazards Allergology and Immunology Medical University of Warsaw Warsaw Poland; ^128^ Allergy and Clinical Immunology Department Centro Medico‐Docente La Trinidad Caracas Venezuela; ^129^ Asthma Reference Center ‐ Escola Superior de Ciencias Santa Casa de Misericórdia of Vitória‐Espírito Santo Vitoria Brazil; ^130^ Faculty of Medicine Fundacion Jimenez Diaz, CIBERES Autonoma University of Madrid Spain; ^131^ The Royal National ENT Hospital University College London UK; ^132^ Immunomodulation and Tolerance Group Imperial College London London UK; ^133^ Department of Dermatology Allergy Unit University Hospital of Zurich Zürich Switzerland; ^134^ The Usher Institute of Population Health Sciences and Informatics The University of Edinburgh Edinburgh UK; ^135^ PROMISE Department University of Palermo Palermo Italy; ^136^ Sociedad Paraguaya de Alergia Asma e Inmunologıa Clinica Sisul, Allergy & Asthma Asuncion Paraguay; ^137^ Finnish Meteorological Institute (FMI) Helsinki Finland; ^138^ Department of Pediatrics Division of Allergy, Clinical Immunology and Rheumatology Federal University of São Paulo São Paulo Brazil; ^139^ Kyrgyzstan National Centre of Cardiology and Internal Medicine Euro‐Asian Respiratory Society Bishkek Kyrgyzstan; ^140^ Department of Pediatrics Division of Respiratory Medicine Hospital Nacional de Niños Universidad de Costa Rica San Jose Costa Rica; ^141^ Department of Pediatrics Hospital Nacional de Niños San José Costa Rica; ^142^ Department of Respiratory Medicine University Hospital Olomouc Czech Republic; ^143^ Centre for Inflammation Research Child Life and Health The University of Edinburgh Edinburgh UK; ^144^ Royal Brompton and Harefield NHS Foundation Trust London UK; ^145^ Department of Respiratory Medicine Copenhagen University Hospital Hvidovre Copenhagen Denmark; ^146^ Department of Immunoallergology Faculty of Health Sciences Cova da Beira Covilhã Portugal; ^147^ Imunoalergologia Centro Hospitalar Universitário de Coimbra Coimbra Portugal; ^148^ Allergy Unit Málaga Regional University Hospital‐IBIMA Málaga Spain; ^149^ Allergist Montevideo Uruguay; ^150^ Department of General ORL, H&NS ENT‐University Hospital Graz Medical University of Graz Graz Austria; ^151^ Pneumology and Allergy Department CIBERES Clinical & Experimental Respiratory Immunoallergy, IDIBAPS University of Barcelona Barcelona Spain; ^152^ Department of Social Medicine Health Planning Unit Faculty of Medicine University of Crete Crete Greece; ^153^ Institute of Pathophysiology and Allergy Research Center of Pathophysiology, Infectiology and Immunology Medical University of Vienna Vienna Austria; ^154^ Universidade Federal dos Pampa Uruguaiana Brazil; ^155^ Faculty of Medicine Vilnius University Institute of Clinical Medicine & Institute of Health Sciences Vilnius Lithuania; ^156^ Department of Lung Diseases and Clinical Immunology University of Turku Turku Finland; ^157^ Department of Chest Medicine Centre Hospitalier Universitaire UCL Namur Université Catholique de Louvain Yvoir Belgium; ^158^ Unit of Geriatric Immunoallergology University of Bari Medical School Bari Italy; ^159^ Division of Allergy and Immunology Department of Pediatrics Faculty of Medicine Siriraj Hospital Mahidol University Bangkok Thailand; ^160^ Department of Otorhinolaryngology, HNO‐Klinik Universitätsklinikum Düsseldorf Germany; ^161^ Nova Southeastern University, Fort Lauderdale Florida USA; ^162^ Department of Occupational Diseases and Environmental Health Nofer Institute of Occupational Medicine Lodz Poland; ^163^ Department of Otolaryngology Yong Loo Lin School of Medicine National University of Singapore Singapore Singapore; ^164^ Department of Medicine, Clinical Immunology and Allergy McMaster University Hamilton Ontario Canada; ^165^ Department of Paediatrics Prince of Wales Hospital The Chinese University of Hong Kong Shatin, New Territories Hong Kong China; ^166^ Department of Pulmonology Celal Bayar University Manisa Turkey; ^167^ The Allergy and Asthma Institute Islamabad Pakistan; ^168^ Universidad Católica de Córdoba Universidad Nacional de Villa Maria Argentina; ^169^ Department of Otolaryngology Head and Neck Surgery Beijing TongRen Hospital Beijing China; ^170^ Respiratory and Allergic Diseases University Clinic Golnik Slovenia; ^171^ Department of Clinical Immunology Wrocław Medical University Wroclaw Poland; ^172^ International Primary Care Respiratory Group IPCRG Aberdeen Scotland; ^173^ European Academy of Paediatrics (EAP/UEMS‐SP) Brussels Belgium; ^174^ Personalized Medicine, Asthma and Allergy Humanitas Clinical and Research Center IRCCS Rozzano Milano Italy; ^175^ Department of Allergy Al‐Rashed Allergy Center Kuwait City Kuwait; ^176^ International Airway Research Center First Affiliated Hospital Guangzou Sun Yat‐sen University Guangzhou China; ^177^ Division of ENT Diseases Department of ENT Diseases CLINTEC Karolinska Institutet Karolinska University Hospital Stockholm Sweden; ^178^ Research Center for Anaphylaxis (ORCA) Odense Denmark; ^179^ National Heart and Lung Institute Royal Brompton Hospital & Imperial College London UK; ^180^ Woolcock Emphysema Centre and Sydney Local Health District Glebe New South Wales Australia; ^181^ Foundation for the Development of Medical and Biological Sciences (Fundemeb) Cartagena Colombia; ^182^ NOVA Medical School CEDOC Comprehensive Health Research Center (CHRC) Lisboa Portugal; ^183^ SLaai, Sociedad Latinoamericana de Allergia Asma e Immunologia Branquilla Colombia; ^184^ Equipe EPAR ‐ IPLESP Sorbonne Université Paris France; ^185^ EUFOREA Brussels Belgium; ^186^ Allergy Unit CUF Porto Portugal; ^187^ Center for Rhinology and Allergology Wiesbaden Germany; ^188^ Sach´s Children and Youth Hospital Södersjukhuset Stockholm Sweden; ^189^ Faculty of Medicine Institute of Clinical Medicine Clinic of Chest Diseases and Allergology Vilnius University Vilnius Lithuania; ^190^ Department of Respiratory and Sleep Medicine Royal Prince Alfred Hospital Sydney Australia; ^191^ Faculty of Medicine Institute of Clinical Medicine University of Oslo Oslo Norway; ^192^ Department of Pediatrics Section of Allergy and Immunology UP‐PGH Manila Philipinnes; ^193^ Serbian Association for Asthma and COPD Belgrade Serbia; ^194^ Clinical & Experimental Respiratory Immunoallergy IDIBAPS CIBERES University of Barcelona Spain; ^195^ Alfred Health, Melbourne Victoria Australia; ^196^ Department of Dermatology and Allergy Centre Odense Research Center for Anaphylaxis (ORCA) Odense University Hospital Odense Denmark; ^197^ Faculty of Medicine Institute of Immunology University of Coimbra Coimbra Portugal; ^198^ Faculty of Medicine ICBR ‐ Coimbra Institute for Clinical and Biomedical Research CIBB University of Coimbra Coimbra Portugal; ^199^ Institut de Recerca Sant Joan de Déu Barcelona Spain; ^200^ Rappaport Faculty of Medicine Technion‐Israel Institute of Technology Haifa Israël; ^201^ Allergy and Clinical Immunology Imperial College London London UK; ^202^ Institute of Cinical Medicine University of Copenhagen Copenhagen Denmark; ^203^ University Hospital Centre Covilhã Portugal; ^204^ Faculty of Medicine University of Coimbra Coimbra Portugal; ^205^ Terveystalo Allergy Clinic Turku Finland; ^206^ Otolaryngology Beijing Institute of Otolaryngology Beijing China; ^207^ ALL‐MED Medical Research Institute Wroclaw Poland

**Keywords:** allergic rhinitis, asthma, immunotherapy, precision medicine

## 1 INTRODUCTION

Allergen immunotherapy (AIT), the gradually increasing repeated administration of high doses of allergens to allergic patients, offers the potential for immune tolerance against reactions to the natural exposures to specific allergens. AIT may lead to the long‐lasting remission of allergic symptoms and is the only disease‐modifying intervention in IgE‐mediated allergic respiratory diseases.

This Pocket Guide was developed by an ARIA and EAACI joint study group from a background paper of the ARIA‐MASK study group and from the EAACI guidelines on allergen immunotherapy.

Bousquet J, Pfaar O, Togias A, et al. (2019). ARIA Care pathways for allergen immunotherapy. Allergy 2019; 74: 2087–2102.

Agache, Lau S, Akdis CA, et al. EAACI guidelines on allergen immunotherapy: house dust mite‐driven allergic asthma. Allergy, 2019;74:855‐73.

AIT is a proven therapeutic option for the treatment of allergic rhinitis, conjunctivitis, and/or asthma using sublingual (SLIT) or subcutaneous (SCIT) routes.

However, AIT is more expensive than symptomatic treatments for allergic diseases (excluding biologicals). It is justified (i) in patients with rhinitis otherwise uncontrolled by symptomatic treatment or (ii) as an add‐on to regular asthma treatment in controlled or partially‐controlled asthmatic patients sensitised to house dust mites aiming to decrease asthma exacerbations, rescue and controller medication, and to improve quality of life.

Care pathways are structured multi‐disciplinary care plans detailing the key steps of patient care. They promote the translation of guideline recommendations to their application in clinical practice.

Although many international and national AIT guidelines have been produced, this is the first care pathway for AIT.

This pocket guide applies to sublingual (SLIT) and sub‐cutaneous (SCIT) immunotherapy for allergic rhinitis.

**FIGURE 1:**
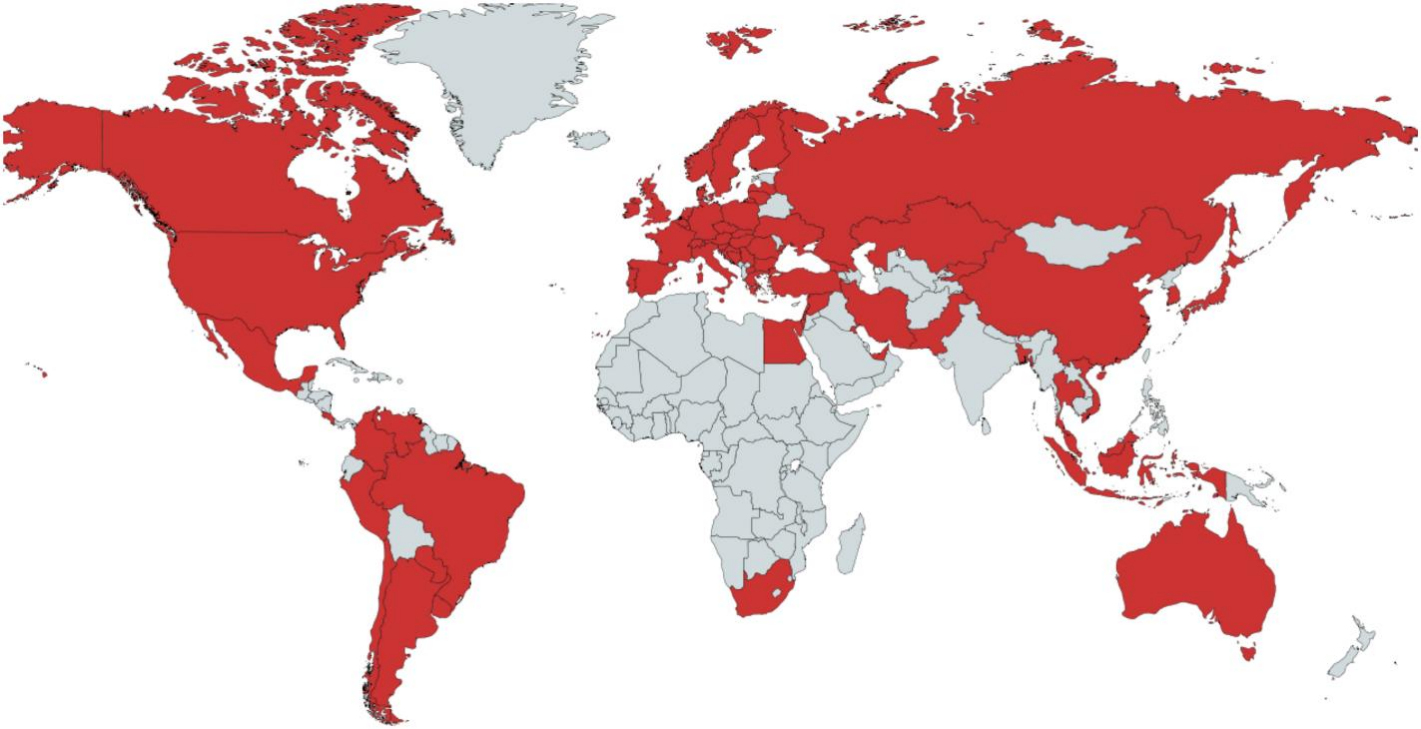
Countries with Pocket Guide members

It has been revised by members from 65 countries (Figure 1).

## 2 ALLERGENS TO BE ADMINISTERED

The decision to prescribe AIT should be based on relevant symptoms during allergen exposure, demonstration of sensitisation to the relevant allergens, and availability of good‐quality extracts with proven efficacy and safety.

Some allergen extracts are approved for marketing in the EU (list in annex) with some others also approved by national health agencies.

For certain products, efficacy and safety have been demonstrated in appropriate clinical studies on adults and children. The extrapolation to untested products, allergens or a different population from the one evaluated in the trial is not appropriate and not in line with current guidelines as there is no class‐effect in AIT.

Both monosensitised and polysensitised patients can be treated. However, in the latter case, the most clinically relevant allergen(s) should be used when symptoms are clearly present with allergen source exposure and when allergy tests confirm clinical findings.

## 3 STRATIFICATION OF ALLERGIC PATIENTS

**FIGURE 2:**
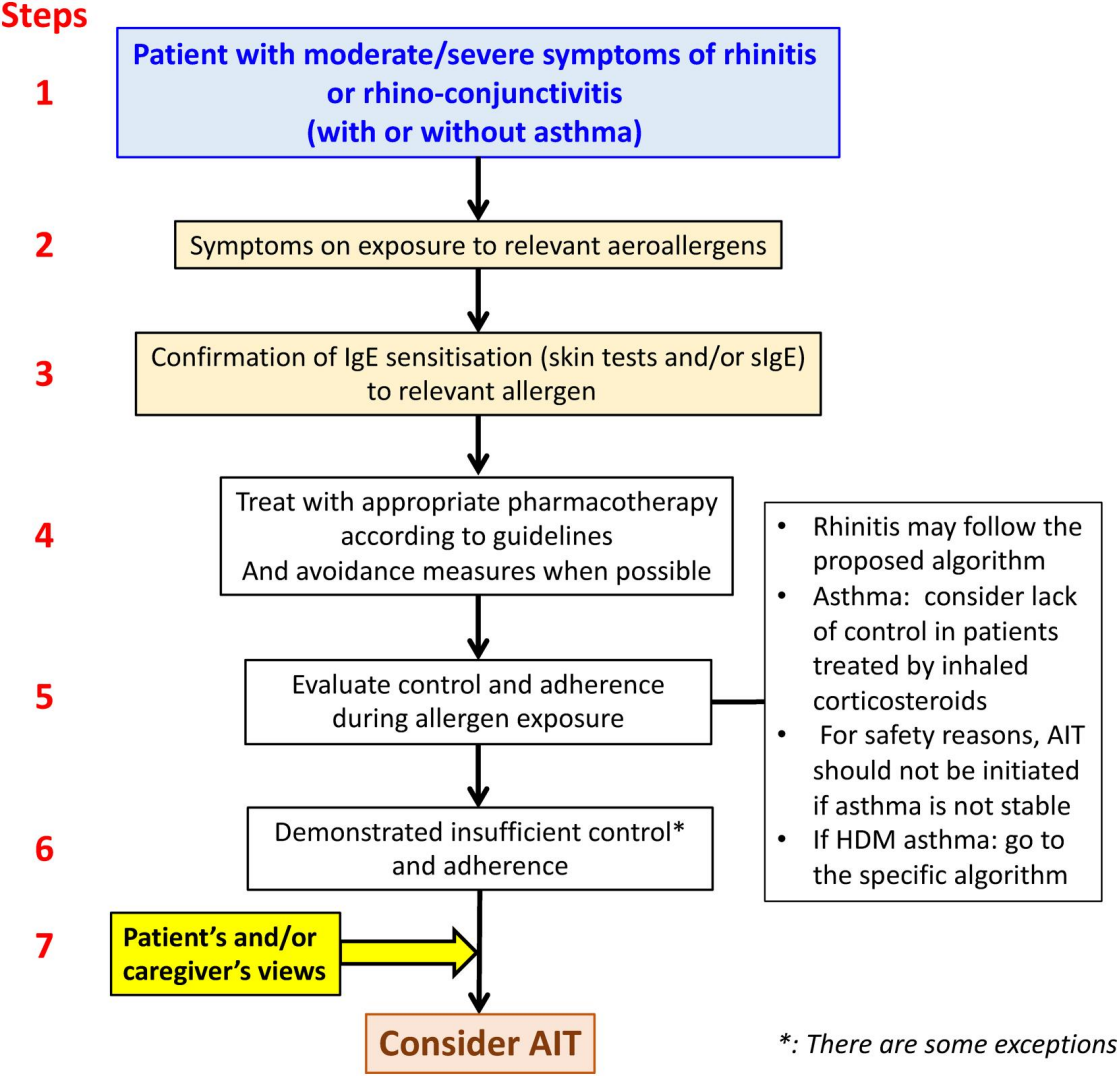
Proposed Flow of Precision Medicine approach in allergic diseases. *examples of exceptions: Thunderstorm‐induced asthma, patient with moderate rhinitis and severe asthma during pollen season

Precision medicine aims at the customisation of healthcare, tailored to the characteristics of each individual patient. The stratification of patients into subpopulations is the basis of clinical decision making (Figure 2).

In allergic diseases, patient stratification is required to:

- Propose the appropriate pharmacotherapy.
- Identify the most suitable candidates for AIT.
- Reduce the amount of time and resources needed to match the right patient to an optimal care management programme.
- Optimise costs as expensive therapeutic interventions are not necessary or suitable for all patients.

Patient stratification may also help to improve the patient's engagement.

4

### 4.1 Precision medicine in the indication of AIT

1. Precise diagnosis with history, skin prick tests and/or specific IgE and, if applicable, component‐resolved in vitro testing. In some cases, where the above‐mentioned diagnostic tools do not allow for precise diagnosis, allergen provocation testing (nasal, ocular and, in some cases, bronchial) may be needed.
2. Proven indications: Allergic rhinitis, conjunctivitis and/or asthma.
3. Symptoms predominantly induced by the relevant allergen exposure.
4. Patient stratification:
  - Poor control of nasal or ocular symptoms despite optimal medications according to guidelines with documented adherence to treatment.
  - Exceptions to requiring optimum symptomatic treatment prior to considering AIT include unacceptable side effects of the medications.
  - Allergic asthma fully controlled under background asthma medication (see EAACI HDM‐AIT GL)
  - However, for partially controlled asthma, HDM‐AIT may facilitate achieving asthma control (see EAACI HDM‐AIT GL)
5. Good clinical documentation of efficacy and safety for the AIT product with relevant trials.
6. The patient's (and caregiver's) views represent an essential component.

### 4.2 Biomarkers

There are currently no in vivo or in vitro biomarkers validated for monitoring the efficacy of AIT although several potential candidates are currently being investigated.

## 5 mHEALTH

Apps can be used:

- To acquire real‐world evidence to confirm the efficacy of AIT in situations where randomised controlled trials are difficult to perform.
- To assess air quality index including pollen exposure and air pollution.
- By physicians and patients for stratification of patients and follow‐up.

## 6 RHINITIS (WITH OR WITHOUT CONJUNCTIVITIS) IN ADOLESCENTS AND ADULTS

The selection of pharmacotherapy and AIT for patients with AR and/or allergic conjunctivitis may be better supported by evidence algorithms to aid patients and healthcare professionals jointly determine the treatment and its step‐up or step‐down strategy depending on rhinitis control (shared decision‐making).

**FIGURE 3:**
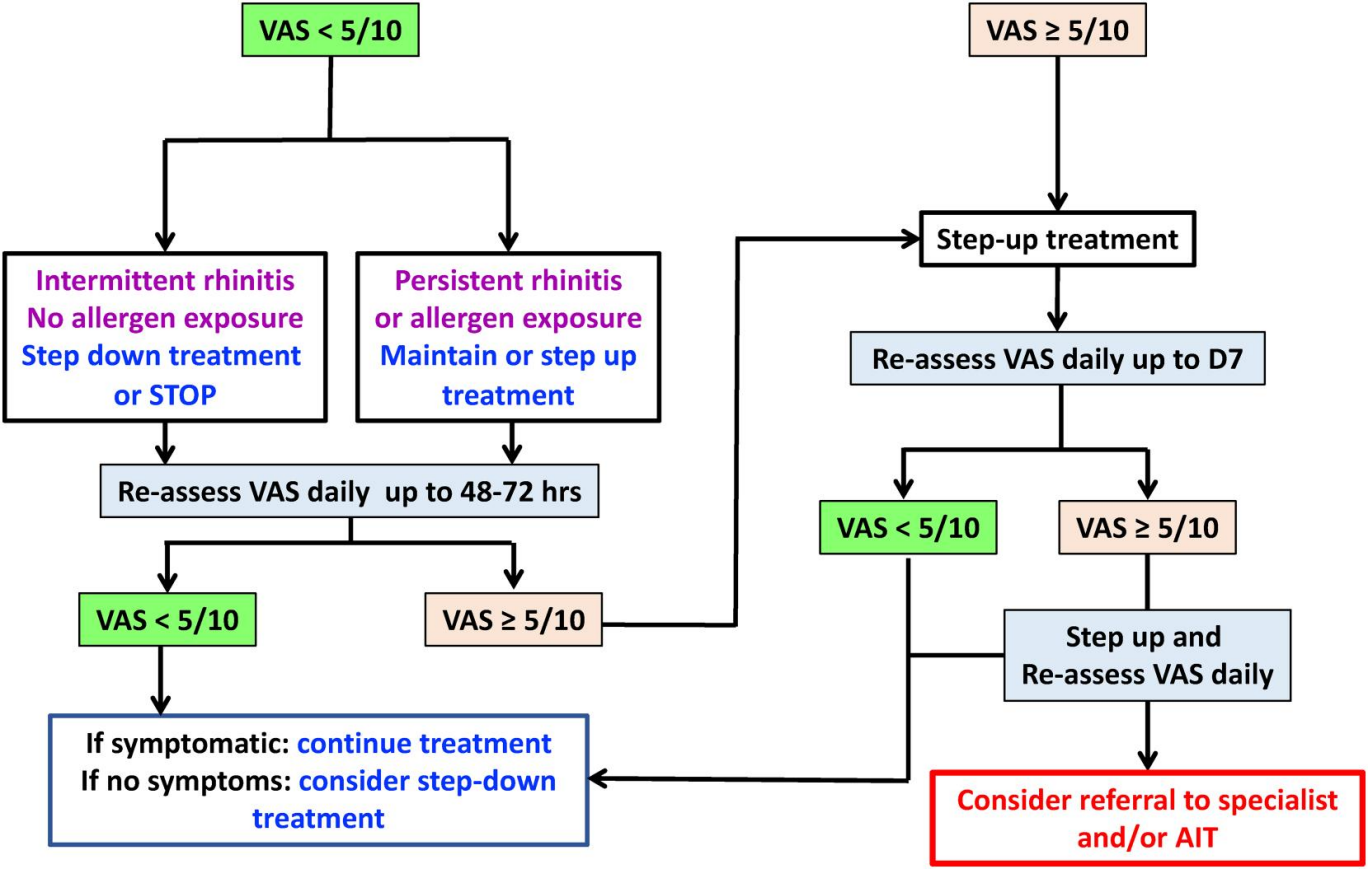
Treatment algorithm using visual analogue scale (VAS) for adolescents and adults AIT, allergen immunotherapy; VAS, visual analogue scale.

A simple algorithm is proposed as an aid for physicians to determine the treatment of their patients (Figure 3).

### 6.1 Treatment algorithm using visual analogue scale (VAS)

In the case of remaining ocular symptoms, add intra‐ocular treatment.

## 7 RHINITIS (WITH OR WITHOUT CONJUNCTIVITIS) IN CHILDREN

AIT is effective, has long‐term beneficial effects after cessation, and may delay or prevent the onset of asthma. AIT can be initiated in children with moderate/severe rhinitis that is not controlled by appropriate medications according to guidelines.

## 8 ASTHMA

An algorithm for HDM‐driven allergic asthma diagnosis and management is proposed by the EAACI guidelines.

For patients with concomitant allergic rhinitis and sensitised to house dust mite—with persisting asthma symptoms despite low‐moderate dose of inhaled corticosteroids—SLIT can be considered, provided FEV1 is >70% predicted.

House dust mite SLIT should initially be considered as an add‐on therapy to controller treatment, and reduction in asthma controllers should be performed gradually under the supervision of a physician.

**FIGURE 4:**
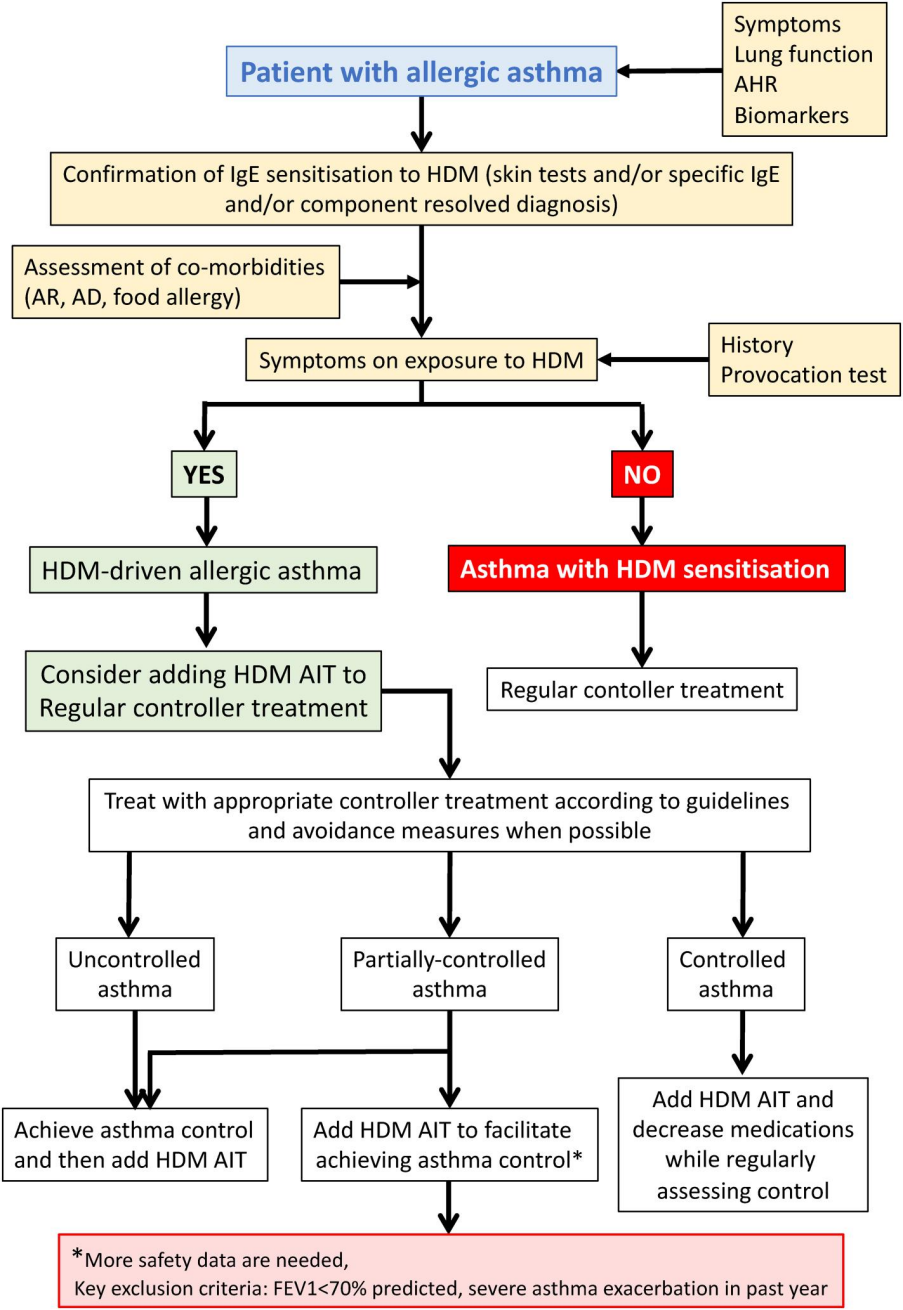
Algorithm for AIT in asthma

Immunotherapy is not indicated for the treatment of acute exacerbations, and patients must be informed of the need to seek medical attention immediately if their asthma deteriorates suddenly (Figure 4).

## 9 MULTIMORBIDITY

One strength of AIT is that it has the potential to control all allergic diseases related to a specific allergen, including rhinitis, conjunctivitis and asthma.

## 10 SAFETY

### 10.1 Subcutaneous immunotherapy (SCIT)

Local reactions: A typical reaction is redness and swelling at the injection site immediately or several hours after the injection. Sometimes, sneezing, nasal congestion or hives can occur.

Systemic reactions: Serious reactions to injections are very rare and require immediate medical attention. Symptoms of an anaphylactic reaction can include swelling in the throat, wheezing or tightness in the chest, nausea and dizziness. The most serious reactions develop within 30 min after the injection, and patients are advised to wait in their doctor's surgery for at least 30 min after an injection. Severe bronchospasm can also occur, especially in patients where asthma is not controlled.

### 10.2 Sublingual immunotherapy (SLIT)

Allergen drops or tablets have a more favourable safety profile than injections. The initial dose should be performed in the doctor's surgery, and patients are advised to remain in the surgery for at least 30 min after administration. Thereafter, SLIT can be administered at home once the first dose has been given under the supervision of a physician.

Allergic reactions: The majority of patients will experience mild local reactions of the oropharyngeal passage. This is usually controlled by predosing with an antihistamine 30 min before the administration of SLIT. Sometimes, sneezing, nasal congestion or hives can occur. Anaphylaxis is rarely described.

In some countries, SLIT tablets include a warning about possible severe allergic reactions, and adrenaline auto‐injectors are routinely recommended. This is not the case in Europe.

## CONFLICT OF INTEREST

IAgache is an Associate Editor Allergy and CTA.

CA reports grants from Allergopharma, grants from Idorsia, Swiss National Science Foundation, Christine Kühne‐Center for Allergy Research and Education, European Commission's Horison's 2020 Framework Programme, Cure, Novartis Research Institutes, Astra Zeneca, scibase, advisory role in Sanofi/Regeneron, grants from Glakso Smith‐Kline, advisory role in scibase.

IA reports personal fees from Hikma, Roxall, Astra Zeneca, Menarini, UCB, Faes Farma, Sanofi, Mundipharma, Bial, Amgen, Stallergenes.

SBA reports grants from TEVA, personal fees from TEVA, AstraZeneca, Boehringer Ingelheim, GSK, Sanofi, Mylan.

VC reports personal fees from ALK, Allergy Therapeutics, LETI, Thermofisher, Merck, Astrazeneca, GSK.

TC reports grants and personal fees from Stallergenes.

PD reports personal fees from ALK‐Abello, Stallergenes‐Greer, Astra Zeneca, GlaxoSmithKline, Mylan, Sanofi.

SD reports personal fees and non‐financial support from ALK Abello, personal fees from Adiga, Biomay, Allergopharma, Anergis, Allergy Therapeutics.

TH reports personal fees from GSK, Mundipharma, Orion Pharma.

SH reports other from ALK‐Abelló, other from ALK‐Abelló.

EH reports personal fees from Sanofi, Novartis, GSK, AstraZeneca, Circassia, Nestlè Purina.

JCI reports personal fees from Faes Farma, Laboratorios Casasco Argentina, Abbott de Ecuador, EuroFarma Argentina.

MJ reports personal fees from ALK‐Abello, Allergopharma, Stallergenes, Anergis, Allergy Therapeutics, Circassia, Leti, Biomay, HAL, during the conduct of the study; personal fees from Astra‐Zeneka, GSK, Novartis, Teva, Vectura, UCB, Takeda, Roche, Janssen, Medimmune, Chiesi,.

LK reports grants and personal fees from Allergopharma, MEDA/Mylan, LETI Pharma, Sanofi, grants from Stallergenes, Quintiles, ASIT biotech, grants from ALK Abelló, Lofarma, AstraZeneca, GSK, Inmunotk, personal fees from Allergy Therapeut., HAL Allergie, Cassella med; and Membership: AeDA, DGHNO, Deutsche Akademie für Allergologie und klinische Immunologie, HNO‐BV, GPA, EAACI.

PK reports personal fees from Adamed, Berlin Chemie Menarini, Boehringer Ingelheim, AstraZeneca, Lekam, Novartis, Polpharma, GSK, Polpharma, Sanofi, teva.

VK reports other from GSK, non‐financial support from Mylan, AstraZeneca, Dimuna, Norameda.

SL reports personal fees from DBV, Sanofi Aventis, Allergopharma, ALK, Nutricia, Bencard.

EM reports personal fees from Sanofi, Novartis, AstraZeneca and Chiesi.

JM reports personal fees and other from SANOFI‐GENZYME & REGENERON, NOVARTIS, ALLAKOS, MITSUBISHI‐TANABE, MENARINI, UCB, ASTRAZENECA, GSK, MSD, grants and personal fees from MYLAN‐MEDA Pharma, URIACH Group.

MO reports personal fees from Hycor Diagnostics, Thermo Fisher Phadia.

YO reports personal fees from Torii Pharmaceutical Co., Ltd., Shionogi Pharmaceutical Co.,Ltd.

OP received research grants from Inmunotek S.L., Novartis and MINECO and has received fees for giving scientific lectures or participation in Advisory Boards from: Allergy Therapeutics, Amgen, AstraZeneca, Diater, GlaxoSmithKline, S.A, Inmunotek S.L, Novartis, Sanofi‐Genzyme and Stallergenes.

NGP reports personal fees from Novartis, Nutricia, HAL, MENARINI/FAES FARMA, SANOFI, MYLAN/MEDA, BIOMAY, AstraZeneca, GSK, MSD, ASIT BIOTECH, Boehringer Ingelheim, grants from Gerolymatos International SA, Capricare.

OP reports grants and personal fees from ALK‐Abelló, Allergopharma, Stallergenes Greer, HAL Allergy Holding B.V./HAL Allergie GmbH, Bencard Allergie GmbH/Allergy Therapeutics, Lofarma, ASIT Biotech Tools S.A., Laboratorios LETI/LETI Pharma, Anergis S.A., Glaxo Smith Kline, grants from Biomay, Circassia, Pohl‐Boskamp, Inmunotek S.L., personal fees from MEDA Pharma/MYLAN, Mobile Chamber Experts (a GA2LEN Partner), Indoor Biotechnologies, Astellas Pharma Global, EUFOREA, ROXALL Medizin, Novartis, Sanofi‐Aventis and Sanofi‐Genzyme, Med Update Europe GmbH, streamedup! GmbH, John Wiley and Sons, AS.

DPreports grants and personal fees from GlaxoSmithKline, personal fees from Menarini, Pliva, Belupo, AbbVie, Novartis, MSD, Chiesi, Revenio, personal fees and non‐financial support from Boehringer Ingelheim, non‐financial support from Philips.

MR is on the Advisory board‐ A. Menarini ‐ Speaker ‐ Astra Zeneca, Novartis, Sanofi, Mylan.

FSRreports speaker and advisory fees from AstraZeneca, Novartis, Sanofi, GSK, Teva and Lusomedicamenta.

GR reports payment to his Institution from Allergo Pharma.

BSreports personal fees from Allergopharma, during the conduct of the study; grants from National Health Programm, grant, personal fees from Polpharma, ASTRA, personal fees from Mylan, Adamed, patient ombudsman, national Centre for Research and Development, Polish Allergology Society.

JS reports grants and personal fees from Sanofi, personal fees from GSK, Novartis, Astra Zeneca, Mundipharma, Faes Farma.

GS reports personal fees from ALK, and leds on the BSACI Rhinitis Guidelines and lead for EUFOREA on Allergic Rhinitis.

PSG reports personal fees from Allergopharma, ALK, grants from Bencard, grants and personal fees from Stallergenes.

JS reports personal fees from Mylan, F2F events.

ATB reports grants and personal fees from Teva, AstraZeneca, GSK Sanofi, Mundipharma, personal fees from Bial, Novartis.

MJTreports grants from European Commission, SEAIC, ISCIII, personal fees from Diater laboratory, Leti laboratory, Aimmune Therapeutics.

MW reports personal fees from ALK‐Abello, Allergopharma, AstraZeneca, Bencard, Genzyme, GlaxoSmithKline, HAL Allergy, LETI, Meda Pharma, Novartis, Sanofi, Stallergenes, Teva.

DW reports other from Optinose, ALK, Sanofi; past Co‐Chair of the Joint Task Force on Practice Parameters of the AAAAI and ACAAI. Second author of a recently published practice parameter on Rhinitis.

MW reports other from Aralez (Medexus), Pediapharm, Pfizer, Astra Zeneca, GSK, Alk.

MZ reports personal fees from Takeda.

TZ reports and Organizational affiliations: Commitee member: WHO‐Initiative “Allergic Rhinitis and Its Impact on Asthma” (ARIA). Member of the Board: German Society for Allergy and Clinical Immunology (DGAKI). Head: European Centre for Allergy Research Foundation (ECARF). Secretary General: Global Allergy and Asthma European Network (GA^2^LEN). Member: Committee on Allergy Diagnosis and Molecular Allergology, World Allergy Organization (WAO).

